# Correction: An initiative to develop capability-adjusted life years in Sweden (CALY-SWE): Selecting capabilities with a Delphi panel and developing the questionnaire

**DOI:** 10.1371/journal.pone.0321980

**Published:** 2025-03-31

**Authors:** The PLOS One Editors

## Notice of Republication

This article [1] was republished on March 17, 2025, to address issues identified post-publication. Please download this article again to view the correct version.

The article’s Data Availability statement is updated to: The data is available upon reasonable request from the Swedish National Data service (SND-ID: 2021–39), by submitting a request for the data at the following DOI: https://doi.org/10.5878/vq4v-dy73.

There was an error in the ethical approval information provided in the third sentence of the first paragraph of the section titled Distribution of capabilities in the population, which incorrectly stated that the study was approved with an advisory statement. The sentence is updated to: The Swedish Ethical Review Authority approved the study with an ordinary statement (Dnr 2019–02848) and participants consented to participate electronically.

## References

[1] Meili KW, Månsdotter A, Sundberg LR, Hjelte J, Lindholm L. An initiative to develop capability-adjusted life years in Sweden (CALY-SWE): selecting capabilities with a Delphi panel and developing the questionnaire. PLoS One. 2022;17(2): e0263231. doi: 10.1371/journal.pone.0263231 35134053 PMC8824323

