# Supplementary material for: An initiative to develop capability-adjusted life years in Sweden (CALY-SWE): Selecting capabilities with a Delphi panel and developing the questionnaire
Source: PLoS One. 2022 Feb 8;17(2):e0263231. doi: 10.1371/journal.pone.0263231 (PMC8824323; doi:10.1371/journal.pone.0263231)
Supplement: S1 File — Screenshots, phrasing with English translation, methods, and results of the web survey and phrasing in Swedish with English translations. (DOCX) [file pone.0263231.s001.docx]

# Supplementary S1 Survey

# Contents

[Supplementary S1 Survey 1](#_Toc94022625)

[Contents 1](#_Toc94022626)

[Survey screenshots 2](#_Toc94022627)

[Survey questions and translations 9](#_Toc94022628)

[Statements 9](#_Toc94022629)

[Sociodemographic background questions 12](#_Toc94022630)

[Material and methods 14](#_Toc94022631)

[Limitations 16](#_Toc94022632)

# Survey screenshots

A version only. For phrasing of all versions and English translation, see the Tables 1 until 7.

Page 1: Consent


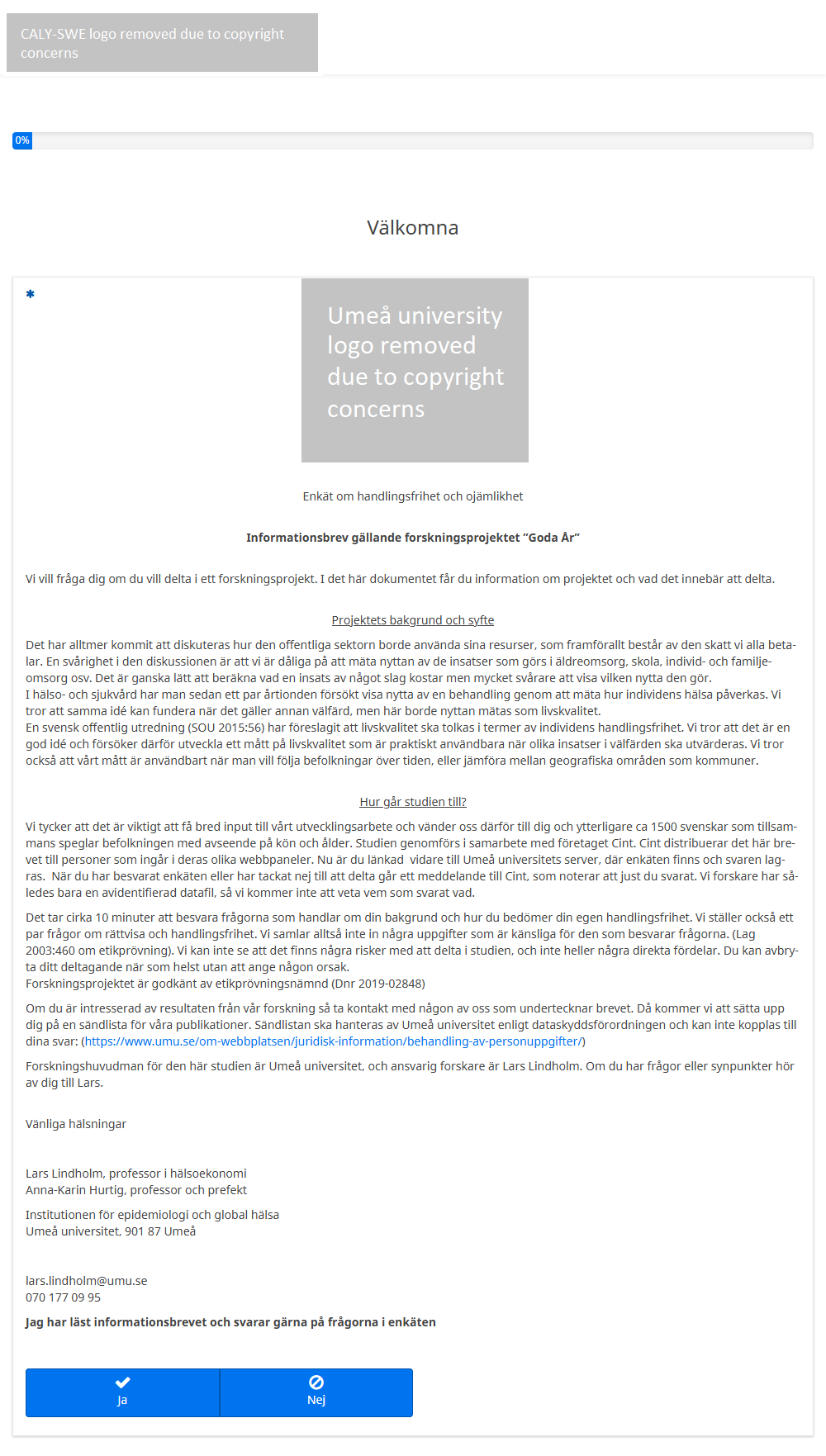


Page 2: Capability statements

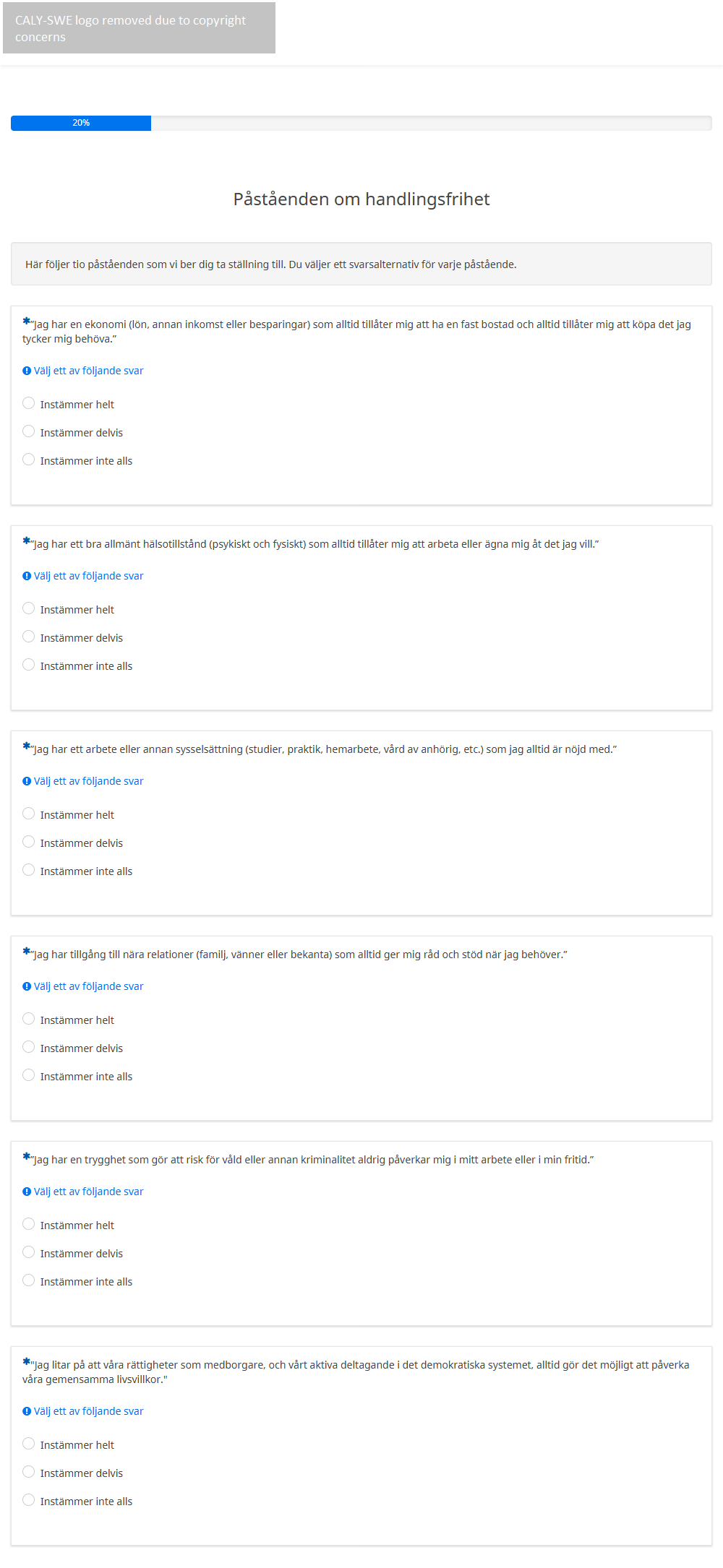


Page 3: Inequality questions

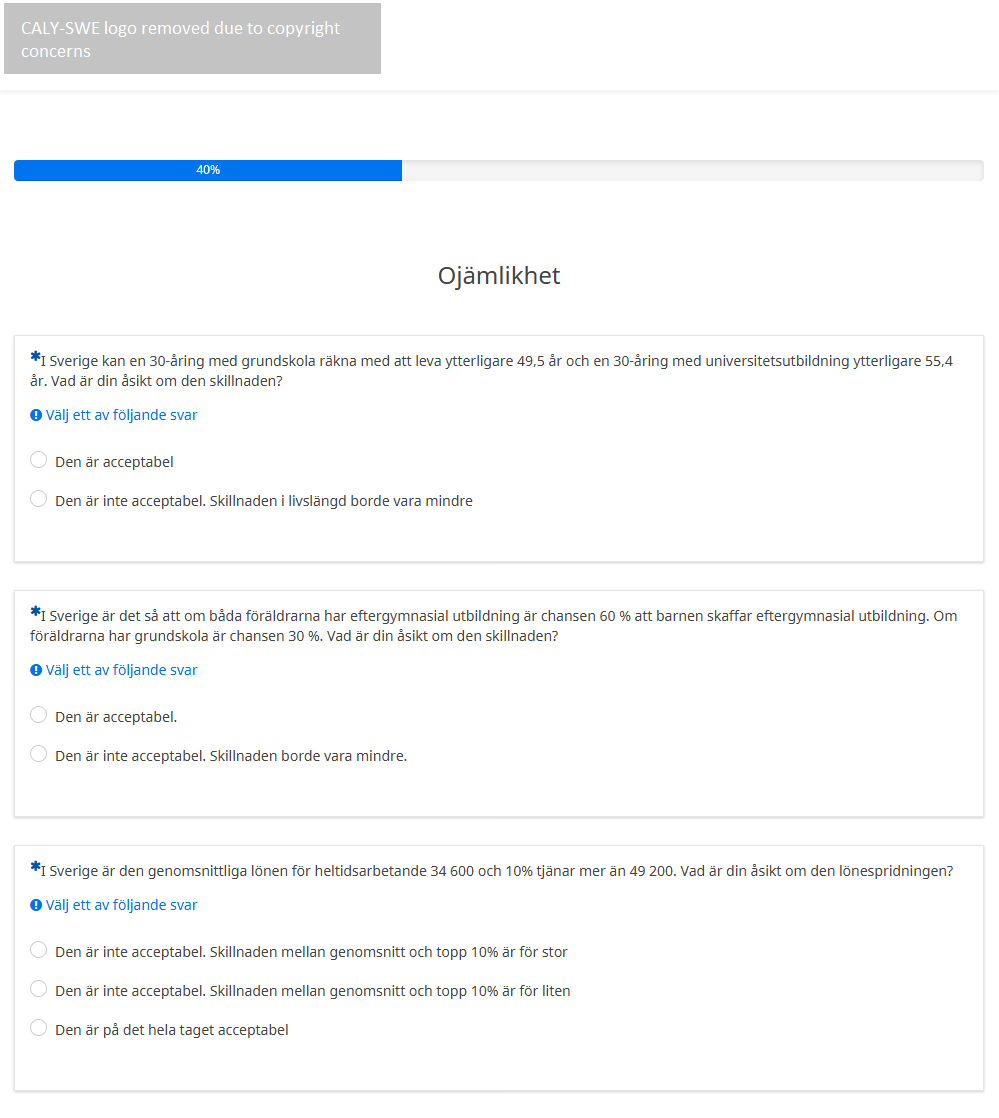


Page 4: Other capability questions (results not reported)

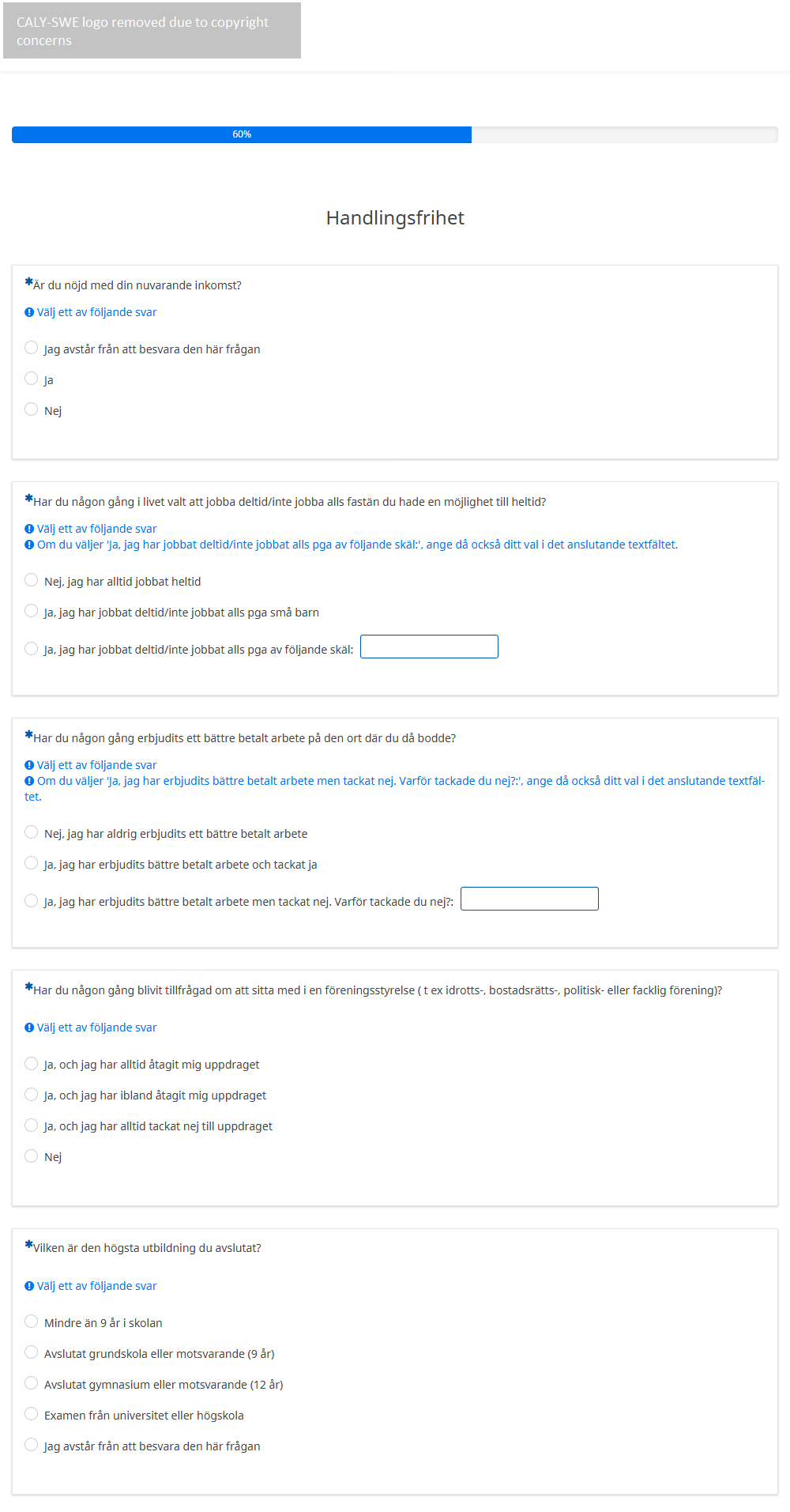


Page 5: Background questions


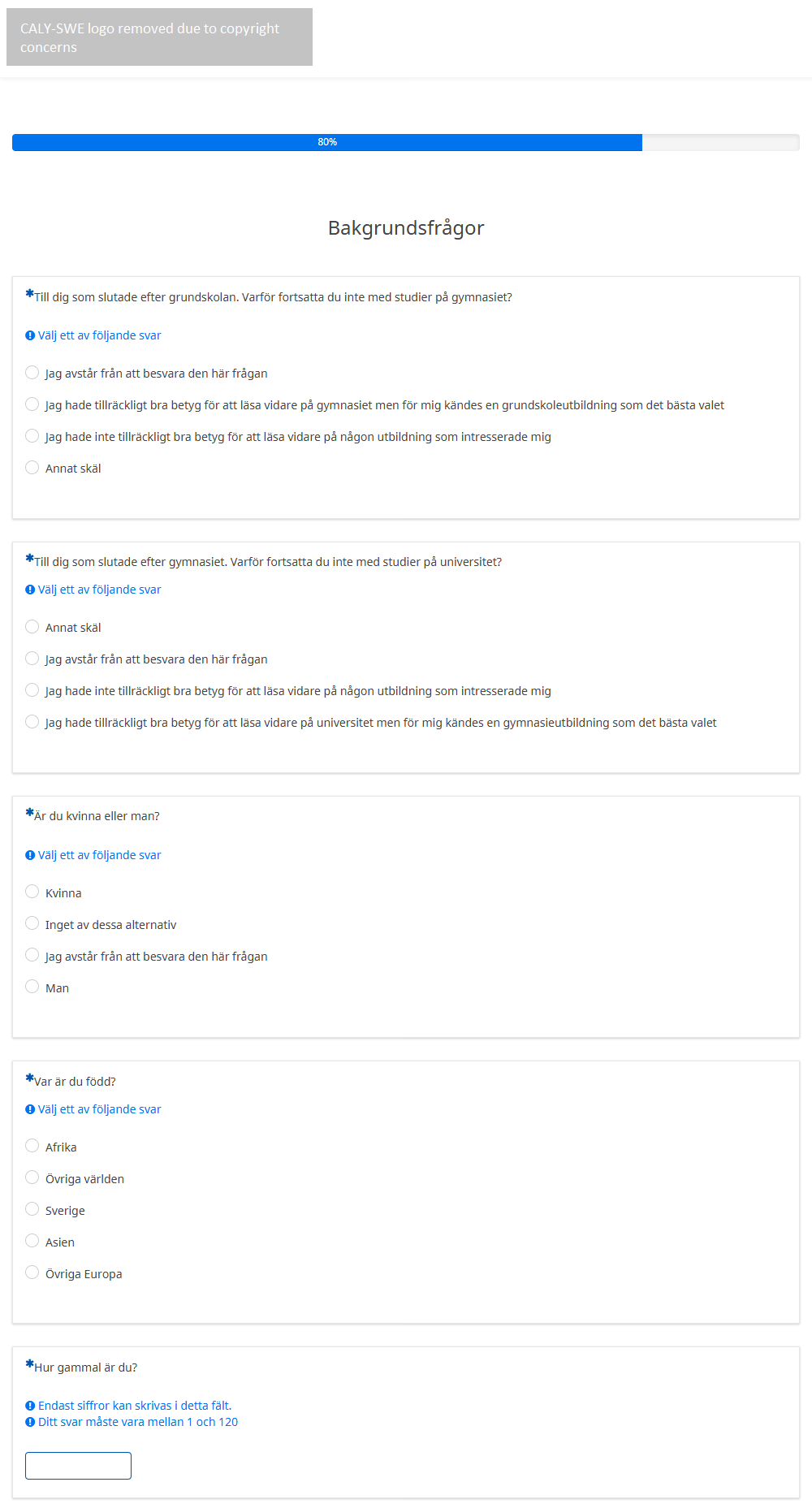


Page 5 continued: Background questions


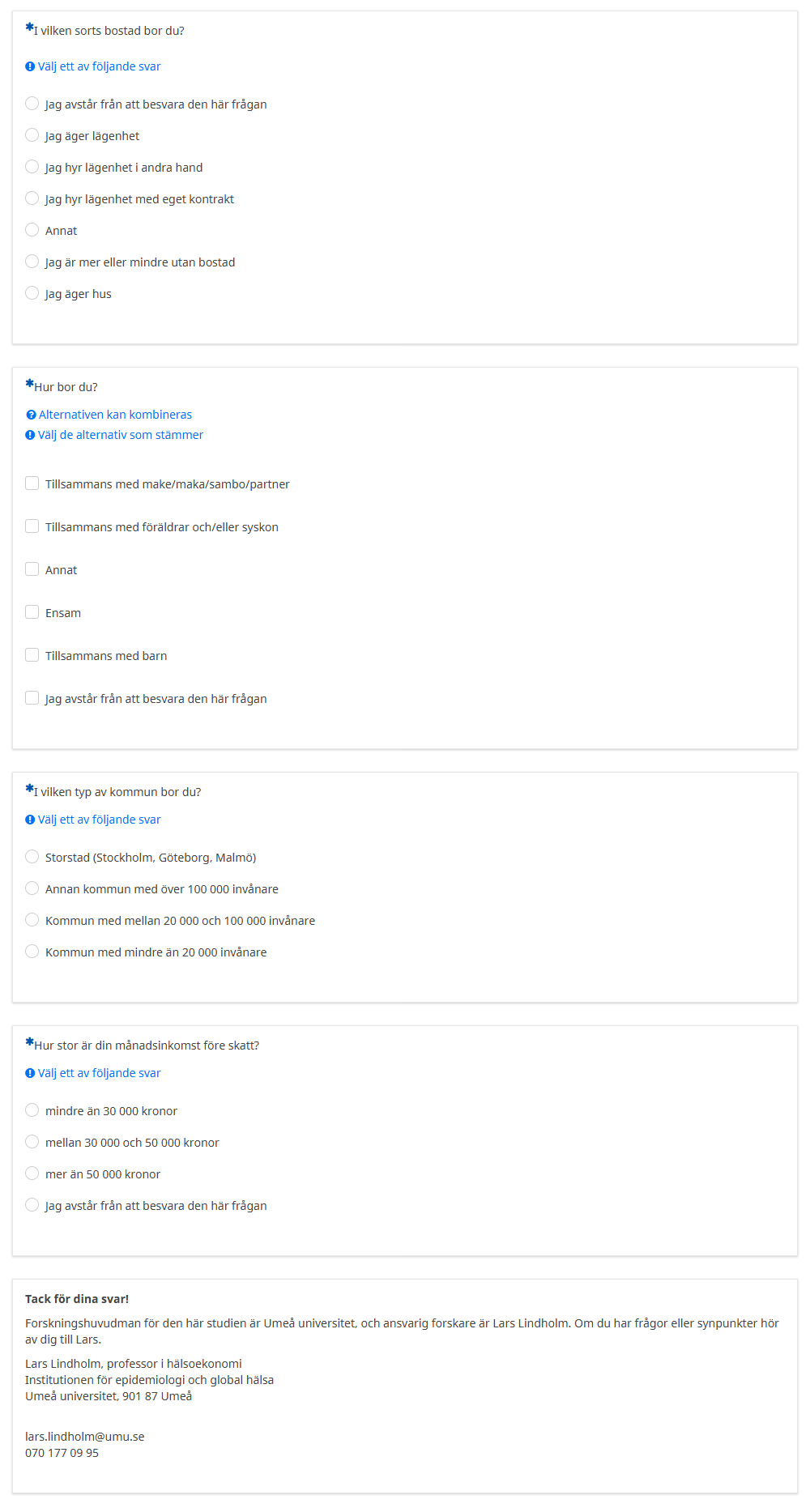


# Survey questions and translations

The translations are not professional and we do not encourage their use.

## Statements

The statements appeared on page 2 of the survey where participants stated for each statement their self-rated capability level.

| **Question Swedish** | **Question English** |
| --- | --- |
| *Här följer tio påståenden som vi ber dig ta ställning till. Du väljer ett svarsalternativ för varje påstående.*   - Instämmer helt - Instämmer delvis - Instämmer inte alls | *Here follow ten statements that we ask you to answer.*   - Agree completely - Agree partially - Not agree at all |

Table 1. Answer translated answer options for self-rated capability.

| **Capability** | **Swedish** | **English** |
| --- | --- | --- |
| *Financial situation & housing* | ”Jag har en ekonomi (lön, annan inkomst eller besparingar) som alltid tillåter mig att ha en fast bostad och alltid tillåter mig att köpa det jag tycker mig behöva." | “I have a financial situation (salary, other income, or savings) that always allows me to have a permanent housing and to buy what I think I need.” |
| *Health* | ”Jag har ett bra allmänt hälsotillstånd (psykiskt och fysiskt) som alltid tillåter mig att arbeta eller ägna mig åt det jag vill.” | ”I have good general health (physical and mental) that allows me to work or to do what I want” |
| *Occupation* | " Jag har ett arbete eller annan sysselsättning (studier, praktik, hemarbete, vård av anhörig, etc.) som jag alltid är nöjd med." | ”I have work or another occupation (studies, internship, household work, care of relatives etc. ) that I am always satisfied with”. |
| *Social relations* | ”Jag har tillgång till nära relationer (familj, vänner eller bekanta) som alltid ger mig råd och stöd när jag behöver." | ”I have access to close relations (family, friends or acquaintances) that always give me advice and support when I need it”. |
| *Security* | ”Jag har en trygghet som gör att risk för våld eller annan kriminalitet aldrig påverkar mig i mitt arbete eller i min fritid.” | ”Risk for violence or other crime does never affect me during my work or my leisure time”. |
| Political & civil rights | "Jag litar på att våra rättigheter som medborgare, och vårt aktiva deltagande i det demokratiska systemet, alltid gör det möjligt att påverka våra gemensamma livsvillkor." | “I trust that my rights as a citizen, and our active participation in the democratic system make it always possible to influence our shared living conditions.” |

Table 2. Swedish capability statements and English translations, version A.

| **Capability** | **Swedish** | **English** |
| --- | --- | --- |
| *Financial situation* | "Jag har en ekonomi (lön, annan inkomst eller besparingar) som alltid tillåter mig att ha en fast bostad och nästan alltid (minst 9 gånger av 10) tillåter mig att köpa det jag tycker mig behöva.  " | “sI have a financial situation (salary, other income, or savings) that always allows me to have a permanent residence and almost always (at least 9 out of 10 times) allows me to buy what I think I need.” |
| *Health* | "Jag har ett bra allmänt hälsotillstånd (psykiskt och fysiskt) som nästan alltid (säg minst 95% av alla dagar) tillåter mig att arbeta eller ägna mig åt det jag vill." | ”I have good general health (physical and mental) that almost always (at least 95% of days) allows me to work or to do what I want” |
| *Occupation* | "Jag har ett arbete eller annan sysselsättning (studier, praktik, hemarbete, vård av anhörig, etc.) som jag nästan alltid är nöjd med. De senaste fem åren har jag minst 90% av tiden varit nöjd med det jag ägnat mig åt." | ”I have work or another occupation (studies, internship, household work, care of relatives etc. ) that I am almost always satisfied with. The last five years I have at least 90% of the time been satisfied with what I devoted myself to.” |
| *Social relations* | "Jag har tillgång till nära relationer (familj, vänner eller bekanta) som nästan alltid (säg minst 9 gånger av 10) ger mig råd och stöd när jag behöver." | ”I have access to close relations (family, friends or acquaintances) that almost always (at least 9 out of 10 times) give me advice and support when I need it”. |
| Security | "Jag har en trygghet som gör att risk för våld eller annan kriminalitet nästan aldrig påverkar mig i mitt arbete eller i min fritid. Uppskattningsvis har jag ändrat mina planer (t ex att jag valt en annan gångväg) vid 10 eller färre tillfällen p.g.a. otrygghet det senaste året." | ”Risk for violence or other crime does almost never affect me during my work or my leisure time. I have changed my plans an estimated 10 or fever times due to unsafety (for example chosen another route for walking) during the last year." |
| Political and civil rights | "Jag litar på att våra rättigheter som medborgare, och vårt aktiva deltagande i det demokratiska systemet, nästan alltid gör det möjligt att påverka våra gemensamma livsvillkor." | “I trust that my rights as a citizen, and our active participation in the democratic system make it almost always possible to influence our shared living conditions.” |

Table 3. Swedish capability statements and English translations, version B.

| **Capability** | **Swedish** | **English** |
| --- | --- | --- |
| *Financial situation* | "Jag har en ekonomi (lön, annan inkomst eller besparingar) som alltid tillåter mig att ha en fast bostad och för det mesta (minst 8 gånger av 10) tillåter mig att köpa det jag tycker mig behöva." | I have a financial situation (salary, other income, or savings) that always allows me to have a permanent residence and mostly (at least 8 out of 10 times) allows me to buy what I think I need.” |
| *Health* | "Jag har ett bra allmänt hälsotillstånd (psykiskt och fysiskt) som för det mesta tillåter (säg minst 90% av alla dagar) mig att arbeta eller ägna mig åt det jag vill." | ”I have good general health (physical and mental) that mostly (at least 9% of days) allows me to work or to do what I want” |
| *Occupation* | "Jag har ett arbete eller annan sysselsättning (studier, praktik, hemarbete, vård av anhörig, etc.) som jag för det mesta är nöjd med. De senaste fem åren har jag minst 75% av tiden varit nöjd med det jag ägnat mig åt." | ”I have work or another occupation (studies, internship, household work, care of relatives etc. ) that I am mostly satisfied with. The last five years I have at least 75% of the time been satisfied with what I devoted myself to.” |
| *Social relations* | "Jag har tillgång till nära relationer (familj, vänner eller bekanta) som för det mesta (säg minst 8 gånger av 10) ger mig  råd och stöd när jag behöver." | ”I have access to close relations (family, friends or acquaintances) that mostly (at least 8 out of 10 times) give me advice and support when I need it”. |
| *Security* | "Jag har en trygghet som gör att risk för våld eller annan kriminalitet väldigt sällan påverkar mig i mitt arbete eller i min fritid. Uppskattningsvis har jag ändrat mina planer (t ex att jag valt en annan gångväg) vid 25 eller färre tillfällen p.g.a. otrygghet det senaste året." | ”Risk for violence or other crime does rarely affect me during my work or my leisure time. I have changed my plans an estimated 25 or fever times due to unsafety (for example chosen another route for walking) during the last year. " |
| Political and civil rights | "Jag litar på att våra rättigheter som medborgare, och vårt aktiva deltagande i det demokratiska systemet, för det mesta gör det möjligt att påverka våra gemensamma livsvillkor." | “I trust that my rights as a citizen, and our active participation in the democratic system make mostly possible to influence our shared living conditions.” |

Table 4. Swedish capability statements and English translations, version C.

| **Question Swedish** | **Question English** |
| --- | --- |
| *I Sverige kan en 30-åring med grundskola räkna med att leva ytterligare 49,5 år och en 30-åring med universitetsutbildning ytterligare 55,4 år. Vad är din åsikt om den skillnaden?*   - Den är acceptabel - Den är inte acceptabel. Skillnaden i livslängd borde vara mindre | *In Sweden a 30-year-old person with elementary schooling can expect to live additional 49.5 years, and a 30-year/old person with university education can expect additional 55.4 years. What is your opinion about this difference?*   - It is acceptable - It is not acceptable. The difference in live expectancy should be smaller |
| *I Sverige är det så att om båda föräldrarna har eftergymnasial utbildning är chansen 60 % att barnen skaffar eftergymnasial utbildning. Om föräldrarna har grundskola är chansen 30%. Vad är din åsikt om den skillnaden?*   - Den är inte acceptabel. Skillnaden borde vara mindre. - Den är acceptabel. | *If in Sweden both parents have post-high-school education there is a 60% chance that the children get an post-high-school education. If parents have elementary schooling the chance is 30%. What is your opinion about the difference?*   - It is not acceptable. The difference should be smaller - It is acceptable |
| *I Sverige är den genomsnittliga lönen för heltidsarbetande 34 600 och 10% tjänar mer än 49 200. Vad är din åsikt om den lönespridningen?*   - Den är på det hela taget acceptabel - Den är inte acceptabel. Skillnaden mellan genomsnitt och topp 10% är för stor - Den är inte acceptabel. Skillnaden mellan genomsnitt och topp 10% är för liten | *In Sweden the average salary for full-time workers is 34 600 and 10% earn more than 49 200. What is your opinion about the salary difference?*   - It is completely acceptable - It is not acceptable. The difference between the average and the top 10% is too big - It is not acceptable. The difference between the average and the top 10% is too small |

Table 5. Inequality aversion questions.

| **Question Swedish** | **Question English** |
| --- | --- |
| *Är du nöjd med din nuvarande inkomst?*   - Ja - Jag avstår från att besvara den här frågan - Nej | *Are you happy with your current income?*   - Yes - I abstain form answering this question - No |
| - *Har du någon gång i livet valt att jobba deltid/inte jobba alls fastän du hade en möjlighet till heltid?* - Ja, jag har jobbat deltid/inte jobbat alls pga små barn - Nej, jag har alltid jobbat heltid - Ja, jag har jobbat deltid/inte jobbat alls pga av följande skäl: [Free text] | *Have you ever in your life chosen to work part time/ not to work at all even though it was possible to work full-time?*   - Yes, I have worked parttime/not at all because of children - No, I have always worked full-time - Yes, I have worked parttime/not at all due to the following reason [Free text] |
| *Har du någon gång erbjudits ett bättre betalt arbete på den ort där du då bodde?*   - Ja, jag har erbjudits bättre betalt arbete och tackat ja - Nej, jag har aldrig erbjudits ett bättre betalt arbete - Ja, jag har erbjudits bättre betalt arbete men tackat nej. Varför tackade du nej? [Free text] | *Have you ever been offered a better job in a location other than where you lived?*   - Yes, I have been offered a better-paid job and said yes - No, I have never been offered a better-paid job - Yes, I have been offred a better-paid job but declined. Why did you decline? [Free text] |
| - *Har du någon gång blivit tillfrågad om att sitta med i en föreningsstyrelse (t ex idrotts-, bostadsrätts-, politisk- eller facklig förening)?* - Ja, och jag har ibland åtagit mig uppdraget - Ja, och jag har alltid tackat nej till uppdraget - Nej - Ja, och jag har alltid åtagit mig uppdraget | *Have you ever been asked to be a board member in an association (for example sports, housing, or political associations or union)*   - Yes, and I have sometimes accepted - Yes, and I have always declined - No - Yes, and I have always accepted |
| *Till dig som slutade efter grundskolan. Varför fortsatta du inte med studier på gymnasiet?*   - Jag avstår från att besvara den här frågan - Jag hade tillräckligt bra betyg för att läsa vidare på gymnasiet men för mig kändes en grundskoleutbildning som det bästa valet - Annat skäl - Jag hade inte tillräckligt bra betyg för att läsa vidare på någon utbildning som intresserade mig | *If you stopped after elementary. Why did you not go high-school?*   - I abstain from answering this question - I had sufficient grades to go to high school but I preferred an elementary education - Other reason - I did not have sufficient grades to pursue a degree that interested me |
| *Till dig som slutade efter gymnasiet. Varför fortsatta du inte med studier på universitet?*   - Jag avstår från att besvara den här frågan - Annat skäl - Jag hade inte tillräckligt bra betyg för att läsa vidare på någon utbildning som intresserade mig - Jag hade tillräckligt bra betyg för att läsa vidare på universitet men för mig kändes en gymnasieutbildning som det bästa valet | *If you stopped after high school. Why did you not go to university?*   - I abstain from answering this question - Other reason - I did not have sufficient grades to pursue a degree that interested me - I had sufficient grades to continue but I preferred a high school education |

Table 6. Capability questions. Not part of the article, listed for completeness.

## Sociodemographic background questions

Appeared on page 4.

| **Question Swedish** | **Question English** |
| --- | --- |
| *Vilken är den högsta utbildning du avslutat?*   - Mindre än 9 år i skolan - Avslutat grundskola eller motsvarande (9 år) - Avslutat gymnasium eller motsvarande (12 år) - Examen från universitet eller högskola - Jag avstår från att besvara den här frågan | What is your highest education that you finished?   - Less than 9 years in the school - Finished basic schooling or similar (9 years) - Finished high school or similar (12 years) - Degree from university or vocal university - I abstain from answering this question |
|  |  |
| *Är du kvinna eller man?*   - Jag avstår från att besvara den här frågan - Kvinna - Inget av dessa alternativ - Man | *Are you man or woman?*   - I abstain from answering this question - Woman - None of those alternatives - Man |
|  |  |
| *Var är du född?*   - Asien - Övriga världen - Övriga Europa - Afrika - Sverige | *Where are you born?*   - Asia - Rest of the world - Rest of Europe - Africa - Sweden |
|  |  |
| *Hur gammal är du?* | *How old are you?* |
|  |  |
| *Hur stor är din månadsinkomst före skatt?*   - mindre än 30 000 kronor - mellan 30 000 och 50 000 kronor - mer än 50 000 kronor - Jag avstår från att besvara den här frågan | *How big is your monthly income before taxes?*   - Less than 30 000 krona - Between 30 000 and 50 000 krona - More than 50 000 krona - I abstain from answering this question |
|  |  |
| *I vilken sorts bostad bor du?*   - Jag hyr lägenhet i andra hand - Annat - Jag äger hus - Jag hyr lägenhet med eget kontrakt - Jag är mer eller mindre utan bostad - Jag äger lägenhet - Jag avstår från att besvara den här frågan | *In what kind of accommodation do you live?*   - I rent an apartment with a sublet contract - Other - I own a house - I rent an apartment - I am more or less without accomodation - I own an apartment - I abstain from answering this question |
|  |  |
| *Hur bor du? (Multiple choice)*   - Tillsammans med barn - Tillsammans med make/maka/sambo/partner - Jag avstår från att besvara den här frågan - Tillsammans med föräldrar och/eller syskon - Annat - Ensam | How do you live? (Multiple choice)   - Together with children - Together with husband/wife/registered partner /partner - I abstain from answering this question - Together with parents and/or siblings - Other - Alone |
|  |  |
| *I vilken typ av kommun bor du?*   - Storstad (Stockholm, Göteborg, Malmö) - Annan kommun med över 100 000 invånare - Kommun med mellan 20 000 och 100 000 invånare - Kommun med mindre än 20 000 invånare | *In what type of community do you live?*   - Big city (Stockholm, Gothenburg, Malmö) - Other municipality with more than 100 000 residents - Municipality between 20 000 and 100 000 residents - Municipality with less than 20 000 residents |

Table 7. Translated sociodemographic background questions.

# Material and methods

We created three different survey versions: A, B, and C. Each version differed in how the capability statements were phrased (Tables 2-4), while answer options remained unchanged.

Initially a description was displayed to the participants that informed about the purpose of the study. The statements on the perceived level of capabilities were preceded by consent to participate and followed by other capability-related questions about inequality aversion and the difference between capability and functionings, along with questions on age, gender and socioeconomic characteristics. Those questions did not differ between versions. Complete phrasing and English translations can be found in the screenshots and Tables 1-7.

We distributed each version with the six capabilities via the online panel with a targeted sample size of 500. Each sample was quota-sampled to represent the Swedish population structure in terms of age, region, and gender. For education, we sampled according to the panel distribution. Sociodemographic background characteristics of the participants are shown in Table 2.

We excluded answers from participants that stated an age below 18 (ethical concerns) and over 99 (data quality concerns) and used R 4.0 [43] with the boostrap [44] package to analyse the survey answers, perform power calculations, calculate boostrapped confidence intervals, and calculate two sample tests for proportions to asses whether the answer proportions differed between survey versions. With a sample size of 500 and a 5% significance level, a two sample test for proportions can detect a difference of 10% with at least 80% power.

We included totally 1,505 participantsin the the analysis; 497 for the “always” A version, 503 for the B “almost always” version, and 505 for the C “mostly” version. The Swedish Ethical Review Authority approved the study with an advisory statement (Dnr 2019-02848).

Table 2. Participants’ characteristics.

| **Strata** | **Category** | **N** | **(Percent)** |
| --- | --- | --- | --- |
| **Monthly income** | <30k SEK | 805 | (53.5) |
|  | 30k-50k SEK | 486 | (32.3) |
|  | >50k SEK | 93 | (6.18) |
|  | NA | 121 | (8.04) |
| **Gender** | Man | 733 | (48.7) |
|  | None of these | 10 | (0.664) |
|  | Woman | 751 | (49.9) |
|  | NA | 11 | (0.731) |
| **Municipality** | <20k inhabiants | 225 | (14.9) |
|  | 20k-100k residents | 499 | (33.2) |
|  | >100k residents | 332 | (22.1) |
|  | Big city | 449 | (29.8) |
|  | NA | 0 | (0) |
| **Education** | <9 years in school | 33 | (2.19) |
|  | Elementary (9 years) | 117 | (7.77) |
|  | High school (12 years) | 690 | (45.8) |
|  | University/vocational | 653 | (43.4) |
|  | NA | 12 | (0.797) |
| **Place of birth** | Sweden | 1263 | (83.9) |
|  | Africa | 13 | (0.864) |
|  | Asia | 68 | (4.52) |
|  | Other Europe | 127 | (8.44) |
|  | Rest of the world | 34 | (2.26) |
|  | NA | 0 | (0) |
| **Housing** | Rent | 488 | (32.4) |
|  | Owns house | 531 | (35.3) |
|  | Owns apartment | 304 | (20.2) |
|  | homeless | 21 | (1.4) |
|  | Rent sublease | 56 | (3.72) |
|  | Other | 81 | (5.38) |
|  | NA | 24 | (1.59) |
| **Living situation*** | Alone | 409 | (0.272) |
|  | With partner | 838 | (0.557) |
|  | With child | 378 | (0.251) |
|  | With relatives | 115 | (0.076) |
|  | Other | 24 | (0.016) |
|  | NA | 17 | (0.011) |
| **Region name**** | Mellersta Norrland | 57 | (3.79) |
|  | Norra Mellansverige | 134 | (8.9) |
|  | Småland med öarna | 131 | (8.7) |
|  | Stockholm | 315 | (20.9) |
|  | Sydsverige | 221 | (14.7) |
|  | Västsverige | 286 | (19) |
|  | Östra Mellansverige | 258 | (17.1) |
|  | Övre Norrland | 88 | (5.85) |
|  | NA | 15 | (0.997) |
| **Age category** | (1,30] | 335 | (22.3) |
|  | (30,48] | 498 | (33.1) |
|  | (48,64] | 420 | (27.9) |
|  | (64,120] | 252 | (16.7) |
|  | NA | 0 | (0) |
| **Total** |  | 1505 | (1) |

The strata column corresponds to the background questions in the survey, with the exception of ‘region’, which was obtained from the web panel. *Participants could select multiple answers for living situation.

# Limitations

The web panel participants may not have been representative of the Swedish population and our sample may have been too small to truly detect relevant differences between versions.
