# Supplementary material for: An initiative to develop capability-adjusted life years in Sweden (CALY-SWE): Selecting capabilities with a Delphi panel and developing the questionnaire
Source: PLoS One. 2022 Feb 8;17(2):e0263231. doi: 10.1371/journal.pone.0263231 (PMC8824323; doi:10.1371/journal.pone.0263231)
Supplement: S2 File — Finalized CALY SWE phrasings. (DOCX) [file pone.0263231.s002.docx]

**Instruktioner**: Fyll i varje fråga. Kryssa bara i ett av alternativen. Frågorna är påståenden som gäller din nuvarande situation. Kryssa det alternativ som stämmer bäst för dig.

**Hälsa:** ”Jag har ett bra allmänt hälsotillstånd (psykiskt och fysiskt) som nästan alltid (säg minst 95% av alla dagar) tillåter mig att arbeta eller ägna mig åt det jag vill.”

- Instämmer helt
- Instämmer delvis
- Instämmer inte

**Nära relationer:** ”Jag har tillgång till nära relationer (familj, vänner eller bekanta) som ger mig råd och stöd när jag behöver.”

- Instämmer helt
- Instämmer delvis
- Instämmer inte

**Ekonomi och boende:** ”Jag har en ekonomi (lön, annan inkomst eller besparingar) som alltid tillåter mig att ha en fast bostad och för det mesta (minst 8 gånger av 10) tillåter mig att köpa det jag tycker mig behöva.”

- Instämmer helt
- Instämmer delvis
- Instämmer inte

**Sysselsättning:** ”Jag har ett arbete eller annan sysselsättning (studier, praktik, hemarbete, vård av anhörig, etc.) som jag för det mesta är nöjd med. De senaste fem åren har jag minst 75% av tiden varit nöjd med det jag ägnat mig åt.”

- Instämmer helt
- Instämmer delvis
- Instämmer inte

**Säkerhet:** ”Jag har en trygghet som gör att risk för våld eller annan kriminalitet inte påverkar mig i mitt arbete eller i min fritid.”

- Instämmer helt
- Instämmer delvis
- Instämmer inte

**Politiska och medborgerliga rättigheter:** "Jag litar på att våra rättigheter som medborgare, och vårt aktiva deltagande i det demokratiska systemet, gör det möjligt att påverka våra gemensamma livsvillkor."

- Instämmer helt
- Instämmer delvis
- Instämmer inte
