# Supplementary material for: An initiative to develop capability-adjusted life years in Sweden (CALY-SWE): Selecting capabilities with a Delphi panel and developing the questionnaire
Source: PLoS One. 2022 Feb 8;17(2):e0263231. doi: 10.1371/journal.pone.0263231 (PMC8824323; doi:10.1371/journal.pone.0263231)
Supplement: Attachment — Submitted filename: response_to_reviewers_20210423.docx [file pone.0263231.s003.docx]

Dear Reviewer and Editor

We highly appreciate your efforts to review our manuscript and the valuable comments. We carefully considered your feedback and revised our manuscript accordingly.

We specifically shortened the section on the methods and results of the empirical survey considerably by shifting a substantial part to the supplementary. We also revised the section to improve clarity and structure, revised Figure 1 to contain the information in Figure 2 in a more understandable format and removed Figure 2.

We also agree with the reviewers reasoning regarding the name. In 2017 [1] we suggested CALYs as a proper name for measures inspired by Sen’s capability approach and the QALY-approach. All “QALYs” a measure of health-related quality of life and a time dimension in common. The scale for health-related quality of life is anchored between “perfect health” and a death. Drummond also mentions an additional a value premisses: ”Also it should be remembered that the cost-per-QALY ranking does embody a kind of equality, in that a QALY is considered to be worth the same to every individual” [2].

However, in this family of “QALYs”, different methods are used to construct health related quality of life measures. Well-known are EQ5D, SF36/SF6 and HUI, and established methods for estimating weights are TTO and standard gamble. Despite these differences, we label the construct(s) QALY.

Our thinking regarding CALYs follow the same lines. As for QALYs, different methods will be developed, and the content in the measure will certainly varies between countries. Even different methods to assign weights to states will be used. We think capability measures in general can be transformed to CALY, if the states can be properly located on a scale from 0 to 1. Finally, we think equity should be considered in CALYs, for example as in the “sufficient capabilities” approach [3], or our fair-innings model. But other methods might exists or will be developed for trading of equity and efficiency when using CALYs as a measure for resource allocation.

In our paper from 2017, we refer to and describe the content in Mitchell et al. [3]. Of course should Mitchell et al. (and Goranitis et al. [4]) be in the present reference list, and we apologize our carelessness.

Please find our point-by-point response below.

Yours sincerely

The authors

| **Comment** | **Reply** | **Where** |
| --- | --- | --- |
| **Original editor comments** |  |  |
| 1. Please ensure that your manuscript meets PLOS ONE's style requirements, including those for file naming. The PLOS ONE style templates can be found at | We renamed the figure files to start with a capital letter. |  |
| 2. In the Methods section, please provide additional information on participant recrtuiemnt for the online web survey. In particular please  describe any inclusion and exclusion criteria's used. And please provide a justification for the sample size used in your study, including any relevant power calculations (if applicable).  Finally, please provide additional details regarding participant consent. In the ethics statement in the Methods and online submission information, please ensure that you have specified (1) whether consent was suitably informed and (2) what type you obtained (for instance, written or verbal). If your study included minors under age 18, state whether you obtained consent from parents or guardians. If the need for consent was waived by the ethics committee, please include this information. | We included the following sentences in the section **Setting sufficient capability thresholds in Sweden**->**Distribution of capabilities in the population**: Regarding inclusion exclusion criteria “*We excluded answers form participants that stated an age below 18 (ethical concerns) and over 99 (data quality concerns).*”  and regarding power analysis “*We deemed a sample size of 500 as sufficient where with a 5% significance level, a two sample test for proportions detects a 0.1 proportion difference with at least 80% power.*”  We also mention exclusion criteria and power analysis in S1 Survey, under the heading **Material and methods**. The only exclusion criteria we applied were age < 18 or age > 99. In the same section we mention that consent was obtained using the online survey.  Note that beyond age we did not apply any inclusion and exclusion criteria. | Lines 383-384 Lines 388-390  S1 supplement, **Material and methods** |
| 3. In your Data Availability statement, you have not specified where the minimal data set underlying the results described in your manuscript can be found. PLOS defines a study's minimal data set as the underlying data used to reach the conclusions drawn in the manuscript and any additional data required to replicate the reported study findings in their entirety. All PLOS journals require that the minimal data set be made fully available. For more information about our data policy, please see http://journals.plos.org/plosone/s/data-availability. | Thank you for highlighting this. We share PLOS one’s philosophy open access to underlying data. The data is currently under review at the Swedish National Data to be published according to the FAIR principles. We will notify you as soon as the data is published. |  |
| **In-house check April 21** |  |  |
| 1) In the Methods section, please provide additional information on participant recrtuiemnt for the online web survey. In particular please describe any inclusion and exclusion criteria's used. And please provide a justification for the sample size used in your study, including any relevant power calculations (if applicable).  Finally, please provide additional details regarding participant consent. In the ethics statement in the Methods and online submission information, please ensure that you have specified (1) whether consent was suitably informed and (2) what type you obtained (for instance, written or verbal). If your study included minors under age 18, state whether you obtained consent from parents or guardians. If the need for consent was waived by the ethics committee, please include this information. | Thank you for your request. We added “and participants consented electronically to the sentence “The Swedish Ethical Review Authority approved the study with an advisory statement (Dnr 2019-02848) *and participants consented to participate electronically.*”  Also see comment 2. above regarding sample size and inclusion exclusion criteria. | Lines 382-383 |
| 2) Thank you for including your ethics statement on the online submission form: "The Swedish Ethical Review Authority approved the study with an advisory statement (Dnr 2019-02848)."  To help ensure that the wording of your manuscript is suitable for publication, would you please also add this statement at the beginning of the Methods section of your manuscript file. | Thank you for pointing out this issue. We included a modified version of the sentence in the section **Setting sufficient capability thresholds in Sweden**->**Distribution of capabilities in the population**, after the aims. See also 1). | Lines 382-383 |
|  |  |  |
| **Reviewer** |  |  |
| **Set 1** |  |  |
| 1) The appropriateness of calling the set of capabilities a "Capability Adjusted Life Years". The QALY is a broad approach whereby information on health is combined with information on life expectancy. Various questionnaires can be used to assess health. We don't refer to these questionnaires as QALYs. This paper is not the first to suggest CALYs, it has been done by Goranitis et al and by Mitchell et al previously, in relation to ICECAP (incidentally, these authors are not referenced, and hence key literature is missed). So, the approach of CALYs is not novel and a CALY could, in theory be calculated from any capability questionnaire. So I do not feel it is appropriate to refer to one set of capabilities by the term associated with a broader approach. | We agree with your reasoning. And we propose ‘CALY-SWE’ as a proper name. This is what we adopted in the manuscript. In the revision we consistently use CALY-SWE where applicable, but retain ‘CALY’ where we talk about capability-adjusted life years in general.  To give appropriate credit to the previous applications and conceptualizations of CALYs we mention the authors you list and some others we changed the sentence leading up to the aims in the **Introduction**; from “*However, to our best knowledge, the amalgamation of the QALY approach and a set of capabilities covering vital aspects of a good life other than health, is novel*.” to “*While capability-adjusted life years have been suggested and implemented before* [3–6]*, our approach includes equity considerations from the start and is focused on an amalgamation of the QALY approach and a set of capabilities covering vital aspects of a good life other than health.*”  The references consist of Goranitis et al., Mitchell et al., the 2008 report by Lorgelly et al, and and the 2017 ICECAP application by Walters et al. | Through the manuscript Line 155 |
|  |  |  |
| **Set 2** |  |  |
| 2) The wording of the questionnaire and whether it can actually be considered to assess capability at all - I would say it assesses functioning. I am not clear how the authors went from the original set of capabilities (as described by Eriksson) to the wording used in their survey? The phrasing of some capabilities is quite complicated, awkward and restrictive - for example, there will be lots of junior researchers who get paid enough to afford safe and acceptable accommodation, it will not be a lack of money that prevents them from securing long term accommodation, but instead short term contracts and possibly the need to move to take up new jobs. It is difficult for people to respond to questions which combine several different concepts, and I'm not sure how reliably the information could be interpreted. | Thank you for your comment. One of the strongest initiatives from the Delpi-panel was to raise the social problems we have in Sweden due to housing shortage. Some people are literally homeless, many more cramped. Prices have skyrocket, and in the big cities only high-income earners can afford a reasonable housing standard. The Panel also defended the view that a welfare state is committed to implement policies which make it possible to get permanent housing within a reasonable waiting-time, and to a reasonable cost, for all who wants. This should be considered as a capability. If anyone prefer to live with temporary contracts they are free to do so. To give university students temporary contracts during the years they study is a practical arrangement, and would not be interpreted as a limitation of the capability. However, all the young that are forced to stay with parents because they cannot afford own housing suffer a limitation.  To facilitate the interpretation of this capability we plan to have a question about housing in the background section.  In the **Discussion**, we explain why occupation is formulated as a function. One reason is the construction of the welfare state. All who share the benefits of the welfare state (free health care, education etc,) should also share the burdens. | Line 536 |
| I just do not see any value in the second survey. The three samples are small (500) and there are different samples in each. The whole objective and methodology just seem totally bizarre to me. I do not see the contribution of this, and I do not see the value in publishing it. | The logic of our approach is heavily inspired by Alan Williams “Fair Innings” [7]. He describes expected QALYs during lifetime in different social groups, and against this empirical pattern he suggests a fair inning to be, say 70 QALYs. If he had set the fair inning to be 80 years, none of the groups had reached it and if he had set it equal to sixty all would have gotten it. A fair inning in some African country would of course be different from the fair inning in UK, so information about present distribution in the particular setting is helpful.  Our idea is simply to follow in the foot-steeps of Williams. We want to prioritize those worst-off (also referring to Rawls theory of justice), which are those who “not at all” agree to the stated capability. This threshold needs to take the present distribution of a capability into account, exactly as Williams did. If the whole population would be below the threshold, we cannot identify those worst-off. “All” can of course not be worse off. In our data, we found that around 10% were worst-off, and this proportion remained stable between the three samples.  With a sample size of 500, the power to detect a proportion difference of 0.1 between two survey versions, using a two sample test for proportions, is at least 80%, which we deemed adequate for our purposes. We aimed for independent samples because we believe that asking the different capability wordings for each capability in sequence would lead to invalid results as participants might change in how they answer between the wording versions.  The whole section has been shortened, and partly rewritten. We have changed the heading to “*Setting sufficient capabilities thresholds in Sweden*” to better describe the content. The section has also got a new introduction to clarify the logic. | Lines 363-433 |
| Nor, I'm afraid, do I see the logic of using the survey data to inform the wording of the attribute. Surely this is just perpetuating existing inequalities? Discussion around the third objective felt very subjective and speculative. | Mitchell et al. [3] discuss how sufficient capabilities can be defined in ICECAP-O. This instrument has four levels for each capability. Full capability (level 4), a lot of capability (level 3), a little capability (level 2) and no capability (level 1). The threshold is set by choosing a level (i.e. 3) and the threshold need not to be constant over all capabilities. It can vary depending on the real availability of capabilities in a certain context.  In our model the threshold is defined by specifying the extend which a capability is available (always, almost always etc). A person who chose the answer option “fully agree” has reached the “sufficient” level. In similarity with ICECAP-O varies the thresholds for the different capabilities.  Neither ICECAP-O nor our method are “objective”. Rather, to set a threshold is a value-based decision.  We have tried to be transparent, and describe the different aspects which have been taken into account.  How to set concrete threshold for sufficient capabilities so that they are useful in policy-making is not a well-researched topic. We see it thus as a first generation method that we and others try to develop, and they will certainly be refined in the future. | Lines 436-503 |
|  |  |  |
| **Other, representational issues** |  |  |
| - Is the rationale for merging capability and QALYs entirely pragmatic? I don't see any conceptual justification? | We think there are good reasons to define “severity” of a disease as the loss of quality of life times the duration of the state. Seasickness is terrible in the moment, but since you completely recover when you disembark the ferry, it’s not judged to be a serious condition. We think this time dimension is crucial when we judge all capabilities. It matters whether you lack money for a month or suffer poverty for decades. Thus, the inclusion of time is a deliberate, value driven decision.  Additionally, extending the QALY concept to incorporate capabilities seems logical from a welfare theory point of view, as we outline in the section from **Introduction**-> **From welfarism to extra-welfarism and capabilities** section:  In parallel to the development of the capability approach and extra-welfarism based on the shortcomings of welfarism, we see CALYs as an application of the capability approach in line with extra-welfarist ideas to solving problems of resource allocation. There are indeed conceptual points of friction with the capability approach as well, such as the stricter descriptive system that allows for complete orderings, but we think that our concept goes beyond pure pragmatism. | Lines 103-158 |
| - Page 11, line 209: The argument here is confusing. First, I don't understand what is meant by "satisfaction of capabilities". Then, I fail to see how increasing income would not enhance a person's capability? I don't think a very good understanding of sufficiency is demonstrated, and again, references to sufficiency are missing. | Thank you for highlighting this unclarity related to “satisfaction” which may be connotated with “preference”. We changed the wording in this sentence to read “*does not increase capabilities*”. We also changed other uses of “*capability satisfaction*” in the manuscript.  The question if our model perpetuates inequalities is central. In fact, our model is constructed to promote equality. Indeed, increased income enhances capability. However, moving into making priorities between conflicting societal goals, e.g. “the equity-efficiency trade-offs”, one idea could be to put a threshold (say annually 100 000 Euro) that is good enough for giving reasonable opportunities for a flourishing life. Mitchell et al (2016) defines the threshold “as the level of capability at or above which a person’s level of capability wellbeing is no longer a concern for policy”.  On the other end of the income distribution we have people earning only 10 000 Euro annually, certainly not enough for a flourishing life. In our model an increase from 10 000 to say 20 000 would yield increased capabilities in the societal calculus. For instance and individual could change from “Not agree” to “Partially agree” to having the capability. Contrary, an increase from 100 000 to 110 000 would not yield anything in the societal calculus, because already 100 000 leads to the answer “Completely agree” to having the capability. Therefore only polices improving the conditions for those below the threshold would be able to improve the level of capabilities. In particular, policies benefiting those “worst-off” would yield new levels of capabilities. This is a parallel to the fair innings idea by Alan Williams (1997). In resource allocation in health care, priority should be given to those who did not get their fair inning. Our model extends this principal view to all capabilities. | Line 214, 476, 560 |
| - Should we be allocating more resources to those in the poorest health? Does this not depend on their ability to to treatment, and the cost and cost-effectiveness of that treatment? | We agree that resource allocation to people with poor health should be dependent on the cost-effectiveness of the treatment (which ideally would factor in the access to the treatment).  Furthermore, the mainstream normative assumption in cost-effectiveness analysis is health maximization, that is to purely rank treatment according to the cost-effectiveness. This idea has its roots in utilitarianism, and has been repeatedly challenged also within health economics, such as by Wagstaff [8] and Williams [7]. Both argue for abandoning pure health maximization and instead make a trade-off between efficiency and equity.  We can add that pure health maximization is not a reasonable goal in policy-making. In Sweden, and many other countries, equity considerations play a prominent role in public decision-making, and our “model” (CALY-SWE) is intended to facilitate decision-making. Also Sen’s own writing supports  equity considerations and criticize utility or health maximization [9,10] |  |
| Table 4 makes reference to TTO/DCE, but I can't see that this has been discussed in the paper. | Thank you for the comment. We added a sentence that briefly mentions that we plan on using TTO/DCE questions: “*We are also planning to develop a weight tariff using time trade -off and discrete choice evaluation questions.*” | Line 521 |

1.	Månsdotter A, Ekman B, Feldman I, Hagberg L, Hurtig A-K, Lindholm L. We Propose a Novel Measure for Social Welfare and Public Health: Capability-Adjusted Life-Years, CALYs. Appl Health Econ Health Policy. 2017;15: 437–440. doi:10.1007/s40258-017-0323-0.

2.	Drummond MF. Allocating Resources. International Journal of Technology Assessment in Health Care. 1990;6: 77–92. doi:10.1017/S0266462300008941.

3.	Mitchell PM, Roberts TE, Barton PM, Coast J. Assessing sufficient capability: A new approach to economic evaluation. Soc Sci Med. 2015;139: 71–79. doi:10.1016/j.socscimed.2015.06.037.

4.	Goranitis I, Coast J, Day E, Copello A, Freemantle N, Frew E. Maximizing Health or Sufficient Capability in Economic Evaluation? A Methodological Experiment of Treatment for Drug Addiction. Med Decis Making. 2017;37: 498–511. doi:10.1177/0272989X16678844.

5.	Walters K, Frost R, Kharicha K, Avgerinou C, Gardner B, Ricciardi F, et al. Home-based health promotion for older people with mild frailty: the HomeHealth intervention development and feasibility RCT. Health Technol Assess. 2017;21: 1–128. doi:10.3310/hta21730.

6.	Lorgelly P, Lorimer K, Fenwick E, Briggs A. The Capability Approach: developing an instrument for evaluating public health interventions. University of Glasgow; p. 59.

7.	Williams A. Intergenerational Equity: An Exploration of the ‘Fair Innings’ Argument. Health Econ. 1997;6: 117–132. doi:10.1002/(SICI)1099-1050(199703)6:2<117::AID-HEC256>3.0.CO;2-B.

8.	Wagstaff A, Van Doorslaer E, Paci P. On the measurement of horizontal inequity in the delivery of health care. J Health Econ. 1991;10: 169–205.

9.	Sen A. Inequality Reexamined. Oxford: Clarendon Press; 1992.

10.	Sen A. Why health equity? Health Economics. 2002;11: 659–666. doi:https://doi.org/10.1002/hec.762.
