# Supplementary material for: An initiative to develop capability-adjusted life years in Sweden (CALY-SWE): Selecting capabilities with a Delphi panel and developing the questionnaire
Source: PLoS One. 2022 Feb 8;17(2):e0263231. doi: 10.1371/journal.pone.0263231 (PMC8824323; doi:10.1371/journal.pone.0263231)
Supplement: Attachment — Submitted filename: response_to_reviewer_210908.docx [file pone.0263231.s004.docx]

Dear Reviewer and Editor

We would like to extend our appreciation for your efforts in reviewing our manuscript and for providing valuable comments.

We think that the comments and remarks helped us to further improve the manuscript and would like to thank the reviewer.

Please consider our point-by-point response below on the ensuing pages for the changes in the manuscript.

Yours sincerely

Kaspar Walter Meili
Anna Månsdotter
Linda Richter Sundberg
Jan Hjelte
Lindholm

Editor comment:

“Please review your reference list to ensure that it is complete and correct. If you have cited papers that have been retracted, please include the rationale for doing so in the manuscript text, or remove these references and replace them with relevant current references. Any changes to the reference list should be mentioned in the rebuttal letter that accompanies your revised manuscript. If you need to cite a retracted article, indicate the article’s retracted status in the References list and also include a citation and full reference for the retraction notice.”
*Answer:*

We went through all the items in the bibliography and failed to find a rejected publication. We however detected a wrongly cited item: Bibliography item [46] (Fenner F et al., Family and generic names for viruses approved by the International Committee on Taxonomy of Viruses) was cited by mistake on line 368, the correct citation would have been item [31] (Williams A, Intergenerational Equity: An Exploration of the ‘Fair Innings’ Argument). We corrected the issue, the citation now is on line 372.

*Major 1:*

“The manuscript is improved and I think I understand the motivation for the online survey now - the authors seem to set the sufficient level as the level that most people already achieve in the small population surveys. I still don't understand why the same survey was not sent to all 1,500 respondents, with a request for them to indicate whether they can achieve the capability somewhat, mostly or always?? Surely this would have made more sense than seeing how many people from a smaller sub-sample strongly agree that they can achieve the capability somewhat, how many people from a separate and equally small subset strongly agree that they can achieve the capability mostly, etc?? It just seems like an unnecessarily complicated way of getting the information, which breaks the sample down into three small and independent sub-samples.”
*Answer*:

Thank you for the comment and the relevant remark. The intention with not sending the same survey to all the participants was to enable the 3 subsamples that are similar and representative of the Swedish population to answer the statements individually, independently from each other. We wanted to use wording in the statements that is similar to the final version of the questionnaire. With the suggested study design this would have not been possible because

- 1) (if all participants get the 3 versions) the answers to the different statements would not be independent of each other, possibly biased,

or

- 2) (if as the reviewer suggested, a question phrasing in the style of “do you achieve the capability X?”- somewhat, -mostly, -always, would have been used) a phrasing quite dissimilar to the final questionnaire, different answer options would have been necessary to keep a respectably level of face validity and the resulting distribution data would not have the same legitimacy to guide the choice of phrasing for the statements.

*Major 2:*
“Also, I think it could still be clearer that the objective was to match the sufficient level to the existing distribution of achievement. And I STILL think it needs to be acknowledged that this does NOT reflect societal values about what constitutes a good life, but instead risks perpetuating poor performance of social policies or existing inequalities. Where is the incentive for social policies to drive improvements in quality of life if all they need to do is perpetuate existing achievement? Will the sufficient level need to be up-dated over time?”

*Answer*:

Thank you. We agree that the section that outlines the goals of the survey is not enough clearly written, and the part between lines 387 and 401 is rewritten to increase clarity. Among other things, we think a short table (Table 2) can be used to explain the design of the study. We cannot exclude that the sufficient level and phrasing requires to be updated. Indeed, adaptions may be necessary in the future to reflect progress or changed societal values.

The revised pars are on lines 367 to 370, 391 to 392 and 401 to 420.

*Minor 1*:

“Page 9: It is wrong to suggest that ICECAP measures only focus on health, in fact they don't explicitly include health at all. So the work reported here is NOT novel in the sense of moving beyond health outcomes.”

*Answer*: Thank you for your comment, what we write on page 9 is : “While capability-adjusted life years have been suggested and implemented before [1–4], our approach includes equity considerations from the start and is focused on an amalgamation of the QALY approach and a set of capabilities covering vital aspects of a good life relevant other than health.”

For instance, we use time-trade off to establish weights as some QALY approaches do.

We know and even write about measures (ICACAP and other) which have moved beyond health. We did by no means intend to suggest that ICECAP does focus health and apologise. We therefore changed the wording to (on line 158):

“… vital aspects of a good life *relevant for the Swedish policy context*.”

*Minor 2*:

“ I STILL do not believe that the work contributes a sufficient level for a FLOURISHING LIFE (see above) - at no point have the authors asked anyone or considered what constitutes a good life. Instead the work establishes (loosely!!) a rough indication of current achievement on the attributes - the majority of people may have perfectly miserable lives - this is never established or considered.”

*Answer*:

What we argue in the paper, in similarity with other capability approaches, is to give people sufficient capabilities for a flourishing life. Lack of capabilities should not hinder people form living the lives they want. However, sufficient capabilities are not a guarantee for flourishing life. Even if all capabilities met high threshold, there would certainly be people dissatisfied with their lives and living conditions.

The choice of capabilities in our model has been a process in several steps: the governmental investigation, a survey among public health researchers and finally the Delphi-panel with members from different not-for-profit organisations.

When it comes to the level of a capability sufficient for a flourishing life, we try to consider all relevant arguments of which we are aware. In particular, we use input from the Delphi process and the study of distribution of capabilities in the population sample. Alongside these two investigations, we consider Swedish laws and policies, and normative ideas in general, which we judge to be of relevance.

For example, about health we write (on line 463-471 in the revised manuscript):

“**Health:** Based on the input from the Delphi panel, we consider physical and mental health as one of the most (maybe the most) important capabilities. The population survey indicated a pronounced inequality aversion. The threshold should facilitate a high and equitable level of health for everybody. The Healthcare Act [49] and other policy documents [50] point in the same direction. However, “always” having the health to do what one wants may be unrealistic and certainly extremely demanding of resources, and thus complicates the realization of better health for those worst-off, as well as the realization of other capabilities. Therefore, we think the formulation “almost always” is the most reasonable “fair innings”. “

The other thresholds are set using a similar synthesizing reasoning.

*Minor 3:*

Lines 396 to 401 on page 23 - I don't understand what is being said here.

We reworded the section to increase clarity from

“Due to the higher threshold implied by the wording of A (“always”) compared to B (“almost always”) and C (“mostly”), we expected an increased proportion of “completely agree” on C compared to B and on B compared to A. Our intent was to select the wording version with a distribution pattern that fitted normative considerations well. No difference between the versions was an argument for adapting the A version with the shortest and least complex formulation.”

to (now on lines 416 to 420)

“Due to the higher threshold *for a “completely agree” answer* implied by the *stricter* wording of A (“always”) compared to B (“almost always”) and C (“mostly”), we expected an increased proportion of “completely agree” answers *for* C compared to B and *for* B compared to A. *If this expectation would not get support, we assumed that this way of finetuning the phrasing was not meaningful.*”

Please also refer to Major comment 2.

*Minor 4*:

Discussion -Kinghorn HAS used empirical methods to establish a sufficient level of capability well-being for ICECAP-A, but their work (published in Social Science & Medicine) is not acknowledged.

Answer: Thank you, we include now the 2019 Kinghorn paper now in the discussion on line 658 and relate it to our approach on lines 656 to 673:

… “Kinghorn [72] organised deliberative workshops for citizens, and in total took 62 persons part in 8 workshops. One task was to assess the level of sufficient capabilities, defined by ICACAP-A. The level chosen in the workshops was 3,3,3,3,3 where the best possible state is 4,4,4,4,4.

In our model the threshold is defined by specifying the extend which a capability is available (always, almost always, or mostly). A person who chose the answer option “fully agree” has reached the “sufficient” level. Similar to ICECAP-A, the thresholds for the different capabilities may vary. The sufficient level in CALY-SWE level is based on a synthesis of relevant sources such as policies and laws, the Delphi-panel, present distribution, and inequity aversion in the population.

CALY-SWE also explicitly includes special consideration of those “worst-off” (not agree at all). Priority one in policy is to lift those worst-off, priority two to support those who have not reached a sufficient level, but do not belong to those worst-off (partly agree).

All the attempts described above to establish a sufficient level may be quite immature and explorative, and more research is needed in this area. The level for the CALY-SWE measure may, for instance, change over time due to societal progress. “

*Other Changes*:

We also would like to add the results from a pilot study (published in this Journal ref). We suggest the inclusion of the following paragraph after line 509:

“The result from the Panel can be compared with a previous pilot study. The aim of the study was to investigate whether it was possible to rank capabilities included in the Swedish parliament report (35). The participants were researchers or PhD-students, mostly in public health. The main finding was that most of the respondents managed to rank the capabilities included in the set. The results suggest that participants deemed health to be most important, followed by social relations and financial situation. Knowledge, occupation, time, security, political resources, housing, and living environment were ranked lower, and the exact order depends on the metric used to synthesise the individual rankings.”

On line 154 we changed “social care” to “social services” to increase clarity (“Capabilities, as an outcome measure in healthcare, elderly care or social *services*, have gained increasing attention during the last decade”).

We also consistently applied the spelling “worst-off” throughout the manuscript (“instead of worst off”).
